# Systemic Oxidative and Inflammatory Responses to Seasonal Heat Stress in Dairy Cattle: Comparison of Serum and Saliva Biomarkers

**DOI:** 10.3390/ani16121758

**Published:** 2026-06-06

**Authors:** Marta Matas-Quintanilla, Rafael Arana, María del Mar Martínez-Pérez, Noemí Soler-Haro, Rodrigo Muiño Otero, Elena Niceas Martínez Díez, Ana María Gutiérrez

**Affiliations:** 1 BioVetMed Research Group, Department of Animal Medicine and Surgery, Regional Campus of International Excellence “Campus Mare Nostrum”, University of Murcia, Espinardo, 30100 Murcia, Spain; r.arana@um.es (R.A.); mmar.martinezp@um.es (M.d.M.M.-P.); noemi.solerh@um.es (N.S.-H.); agmontes@um.es (A.M.G.); 2 Department of Animal Pathology, Campus Terra-IBADER, University of Santiago de Compostela, 27002 Lugo, Spain; rodrigo.muino.otero@usc.es (R.M.O.); elenaniceas.martinez@rai.usc.es (E.N.M.D.)

**Keywords:** adenosine deaminase, dairy cattle, heat stress, oxidative stress, saliva, serum

## Abstract

Heat stress is a physiological challenge for dairy cattle, linked to oxidative imbalance and immune dysregulation. This study evaluated seasonal heat stress effects on systemic redox and inflammatory status in healthy dairy cows and tested saliva as an alternative diagnostic fluid to serum. A total of 114 clinically healthy Holstein cows were sampled across summer, autumn, and winter (38 cows per season). Environmental exposure was quantified using the temperature–humidity index (THI). Paired saliva and serum samples were analyzed for total oxidant status (TOS), total antioxidant capacity (TAC) and adenosine deaminase (ADA); the oxidative stress index (OSI = TOS/TAC) was calculated. In saliva, TOS, TAC and ADA were significantly lower in summer than in autumn and winter, while salivary OSI remained stable across seasons. In serum, summer was characterized by increased TOS and OSI together with reduced ADA activity, indicating systemic oxidative stress accompanied by diminished ADA-linked immune activation under heat stress. Although salivary markers correlated with THI, saliva did not mirror the systemic oxidative imbalance detected in serum. Under the conditions and biomarkers evaluated, serum provided a more reliable assessment of heat-stress-related redox and immune disturbances in dairy cattle, whereas saliva showed limited utility as a substitute for monitoring.

## Simple Summary

Heat stress reduces dairy cows’ health and productivity by upsetting antioxidant balance and altering immune responses. We evaluated whether these changes are detectable in serum and in saliva, a non-invasive sample useful for field monitoring. A total of 114 healthy Holstein cows were sampled in summer, autumn and winter; environmental exposure was measured with the temperature–humidity index (THI). Paired serum and saliva samples were analyzed for TOS, TAC and ADA; the OSI = TOS/TAC was calculated. In summer, serum showed clear oxidative imbalance (higher TOS and OSI) and lower ADA activity, indicating systemic oxidative stress and altered immune activation. Saliva behaved differently: TOS, TAC and ADA were lower in summer and salivary OSI did not change, so saliva did not consistently reflect the serum changes. Some salivary measures correlated with THI, but variability in saliva flow limits its reliability as a standalone substitute for serum. Serum remains the preferred matrix for monitoring heat-related physiological disturbances; selected salivary markers could complement field assessments if collection and validation are standardized.

## 1. Introduction

Heat stress is a major environmental challenge in dairy cattle, with profound consequences for animal health, welfare, and productivity [1,2]. In lactating cows, high ambient temperature combined with humidity impairs thermoregulation, increases metabolic strain, and reduces feed intake, milk yield, and reproductive efficiency [3,4]. Because high-producing cows generate substantial metabolic heat, they are especially susceptible to thermal load; the physiological effects become evident once environmental conditions exceed the animal’s capacity to dissipate heat [2,5].

Some of the disturbances of homeostasis that need to be defined in order to understand the changes that occur at the organism level with increased heat stress are inflammation and oxidative stress [6]. Inflammation is a fundamental defence mechanism of the immune system, triggered by cellular and tissue injury induced by diverse agents [7]. On the other hand, oxidative stress is defined as an imbalance between oxidants and antioxidants, which can cause damage [8]. In lactating cows, their increase is related to pathologies such as mastitis, metritis, processes such as pneumonia and digestive problems [9], but, in addition, it can be increased by agents such as high temperature [6].

Adenosine deaminase (ADA) is an enzyme closely related to the activation of the immune system [10] with T-lymphocyte and macrophage activity [11] and, therefore, is related to inflammation [12]. In cows with inflammatory pathologies, ADA has higher values compared to control cows in serum and in saliva samples [13].

Total oxidative status (TOS) is used to determine the overall oxidation of the organism [14], the increase of which predisposes the organism to susceptibility to metabolic and infectious problems [9]; meanwhile, total antioxidant capacity (TAC) is responsible for inactivating oxidants, reaching a redox balance and protecting against damage [15]. To relate the last two parameters described, we refer to the oxidative stress index (OSI), which is the ratio of the imbalance between oxidants and antioxidants. This index can be very valuable, as OSI can be a consequence of excessive oxidant production and/or a decrease in the body’s antioxidant defence; therefore, these parameters are strictly independent [16].

Saliva collection is a non-invasive method that does not require specialized staff, but in ruminants, the usefulness of saliva is limited, probably because of the continuous production of saliva in these species and the balance between rumination times [17]. This limitation is further compounded by the potential dilution of biomarkers due to high saliva volume [18], contamination with feed or ruminal contents [19], and variability in composition related to diet and physiological state [20]. Moreover, serum is an invasive method, but it gives better results in inflammatory pathologies in ruminants than saliva [18]. However, the use of saliva as a biomarker fluid in ruminants could be further explored. More recently, salivary heat shock protein 70 (HSP70) has emerged as a promising, highly specific non-invasive biomarker for monitoring environmental thermal stress in ruminants [21].

Heat stress is defined as the sum of external environmental factors, which include ambient temperature, relative humidity, solar radiation and wind speed, acting on the animal causing an increase in body temperature and the activation of physiological and behavioural adjustments [1]. The optimal range in temperature for dairy cattle is between 5 °C and 25 °C, and in relative humidity from 40% to 70% [22].

In order to know the degree of heat stress to which these animals are exposed, the temperature–humidity index (THI) parameter is used, which estimates the magnitude of heat stress [23]. The equation that gives us this index is THI = (1.8 × T + 32) – (0.55 – 0.00555 × RH) × (1.8 × T – 26), where T = ambient temperature (ºC) and RH = relative humidity (%) [24].

It has been found that lactating cows may begin to experience heat stress at a THI as low as 66 [3], although early signs are more commonly reported from values of approximately 68, with more consistent effects observed above 72, depending on production level and environmental conditions [1,5]. These conditions are associated with reductions in milk production, feed intake and reproductive performance [2,4,5]. Specifically, heat stress is associated with decreased milk production, partly driven by reduced feed intake, and alterations in milk composition [4,5]. Reproductive performance is also impaired, with reductions in conception rates and altered estrous expression [1,5]. In addition, heat stress induces metabolic changes, including negative energy balance, that further contribute to decreased productivity [4,5]. 

This study has two objectives: first, to evaluate the impact of seasonal heat stress on healthy dairy cattle by studying serum biomarkers of oxidative and inflammatory status, and second, to assess whether saliva is an analytical biological fluid as useful as serum for analyzing the impact of heat stress.

## 2. Materials and Methods

### 2.1. Experimental Animals

All the experimental procedures were performed in accordance with the Bioethics Committee of the University of Murcia (UMU) (CE 972/2025), the Spanish (RD 53/2013, legal provision no.1337, [25]), and the European Regulation on Animals for Scientific Purposes [26].

A total of 114 healthy Holstein dairy cows, 38 per season, were included in the study. The samples were obtained in summer, autumn and winter from animals located on one farm in the southeast of Spain, Murcia. Spring was not included because environmental conditions in autumn and spring are usually similar in the study region. Sampling was always conducted between 7:30 a.m. and 10:30 a.m.

During the study periods, the environmental conditions were as follows: Summer (July): mean temperature 27.95 ± 0.33 °C and RH 77.16 ± 1.04%; mean THI 79.30. Autumn (October/November): mean temperature 15.22 ± 0.20 °C and RH 62.03 ± 1.76%; mean THI 58.74. Winter (January): mean temperature 11.10 ± 0.20 °C and RH 72.87 ± 0.56%; mean THI 53.49. Temperature and relative humidity were recorded on-farm using a digital thermo-hygrometer (Gulaki, Langfang, China) placed in the shade near the animals before sampling.

Milk yield (mean kg/day) and pregnancy rate (number of pregnancies/numbers of eligible per month, %) were recorded to characterize the productive and reproductive status of the animals during each season.

### 2.2. Environmental and Management Conditions

The animals were housed in open-air pens in which the feeding areas were covered, and shaded areas represented approximately 30–40% of the total pen area. In summer, all the cows were cooled using a Cow-Cooling fan-sprinkler system (model DDF1200 P, DeLaval, Tumba, Sweden) operated 4-5 times daily in the waiting area. The cooling system consisted of low-flow, coarse-droplet sprinklers (1.2–1.4 L/min per sprinkler) operated in cycles of approximately 45 s of sprinkler operation followed by 5 min of continuous ventilation in the waiting area. The estimated water consumption of the sprinkler system was approximately 280–300 L/h. In addition, fans were distributed in the feeding and resting areas. In autumn and winter, no cooling systems were active. The diet remained stable throughout the study, consisting of 60% by-products (broccoli, citrus pulp, beet pulp, and artichoke) and 40% alfalfa, cottonseed, straw, and concentrate.

### 2.3. Sample Collection

Both saliva and blood samples were collected individually from all the animals by the official veterinarian of the farm. Sampling was consistently performed during the morning (approximately between 07:30 and 10:30 h) using the same sampling procedure under similar management and feeding conditions across all evaluated seasons, shortly before feeding. For the saliva samples, 2 cm × 2 cm × 1 cm sponge pieces were used, attached to a Kocher clamp, which were introduced into the mouth, between commissures, of the dairy cows, for 1 min until fully saturated. They were then inserted into specifically designed tubes for saliva collection (Salivette tubes, Sarstedt, Nümbrecht, Germany).

The blood samples were obtained after saliva sampling by coccygeal venipuncture using a Vacutainer^®^ system with an 18G (1.2 × 40 mm) needle, a holder, and red-top tubes without anticoagulant for serum collection.

The samples were kept with cold accumulators in a portable refrigerator from collection until centrifugation at the laboratory, which was performed within 2–3 h, at 3000× *g* for 10 min. After centrifugation, the saliva samples were visually inspected to discard any samples with visible blood or dirt contamination.

Subsequently, the serum and saliva samples were transferred into 1.5 mL tubes and stored at −80 °C until analysis.

### 2.4. Analytic Procedures

#### 2.4.1. Total Antioxidant Capacity Level Quantification

TAC was measured in both biological fluids according to the method of the ferric reducing ability of plasma (FRAP) [27]. The assay was performed using 36 μL of sample and 270 μL of FRAP reagent at 37 °C. No dilutions were required in any biological fluid. The absorbance of the reaction was measured at 593 nm in a microplate reader (Spectro Star Nano, BMG Labtech, Ortenberg, Germany). Variation coefficients were lower than 9%, and the detection limit was 0.80 μM/L Trolox equivalent [13].

#### 2.4.2. Total Oxidant Status and Oxidative Stress Index

To measure TOS, a commercial assay (PierceTM Quantitative Peroxide assay, Thermo Scientific, Rockford, IL, USA), previously optimized for dairy cow saliva and serum, was used [13]. The assay for saliva samples required 120 μL of the sample and 100 μL of the working buffer; in the case of serum, 60 μL of the sample and 160 μL of the working buffer were necessary. The absorbance of the reaction after 15 min of incubation at room temperature was quantified at 595 nm in a microplate reader (Spectro Star Nano, BMG Labtech, Ortenberg, Germany). Coefficients of variation were lower than 7% for the intra-assay precision and lower than 10% for the inter-assay precision in both biological fluids. The detection limit was 0.5 μM/L equivalents of peroxidase [13]. OSI was calculated by means of TOS/TAC [16].

#### 2.4.3. Adenosine Deaminase Activity Measurement

Total ADA activity in both the saliva and serum samples was quantified using a previously validated adaptation of a commercial automatized human assay (BioSystems S.A., Barcelona, Spain) in a 96-well microplate [13]. The cow saliva samples required a 1:8 dilution, whereas no dilution was necessary in the serum samples. The method consisted of the reaction of 50 μL of the sample and 200 μL of the ADA reagent. The absorbance value of the reaction was monitored at 340 nm each 30 s for 3 min using a microplate reader (Spectro Star Nano, BMG Labtech, Ortenberg, Germany).

ADA activity was obtained by calculating the maximum decrease in the absorbance per minute (Δabs/min × 3333 = U/L). The variation coefficient of the intra-assay was lower than 11%, and the detection limit was 9.3 U/L [13].

### 2.5. Statistical Analysis

Data analysis was carried out with statistic software GraphPad Prism (version 10.6.0). A *p*-value < 0.05 was considered statistically significant for all analyses.

Data distribution was assessed using the Shapiro–Wilk test and homogeneity of var-iances using Bartlett’s test. Variables that did not fulfil the homoscedasticity criteria independently of the data distribution (TOS, TAC, OSI, serum ADA, milk yield and pregnancy rate) were analyzed using Brown–Forsythe and Welch ANOVA tests followed by Dunnett’s T3 multiple comparisons test. Variables fulfilling the assumptions of normality and homoscedasticity (salivary ADA and THI) were analyzed using ordinary one-way ANOVA followed by Tukey’s multiple comparisons test. Effect sizes for the statistically significant comparisons observed were estimated as Cohen’s d together with their corresponding 95% confidence intervals using Jamovi statistical software (version 2.4.12). Cohen’s d values were interpreted according to Cohen’s conventional thresholds [28] as small (0.2), medium (0.5), and large (0.8) effects. 

Associations between THI and the different biomarkers were assessed using Spearman’s rank correlation coefficient.

## 3. Results

### 3.1. The Heat Stress Analysis

**Figure 1:**
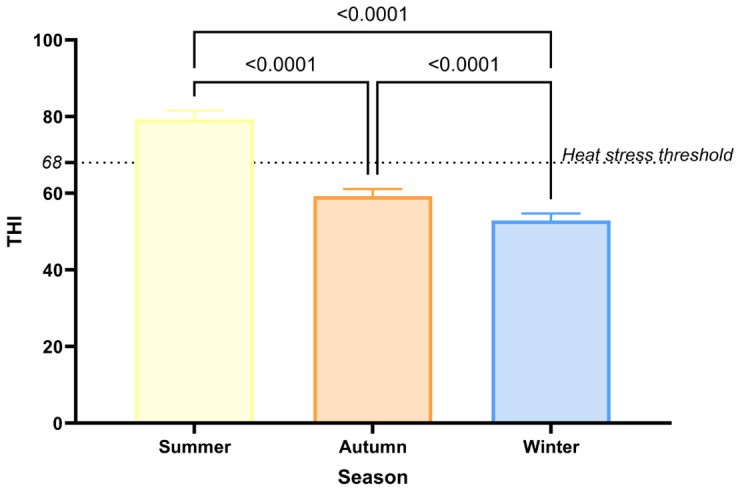
The values of the temperature–humidity index (THI) during the sampling days of the healthy dairy cows in summer, autumn and winter. The graph shows the means (boxes) and standard errors of the means (error bars). Horizontal brackets represent statistically significant differences between the groups; the level of significance is reported.

Regarding the environmental conditions on the days when samples were collected from the dairy cattle, the THI calculated showed a wide gap between summer, which presented the statistically highest values, and the rest of the seasons (Figure 1). Significant differences were also observed between autumn and winter. Effect sizes for the comparisons between autumn and summer, autumn and winter, and summer and winter were 9.74 (95% CI = 8.38 to 11.09), 3.08 (95% CI = 2.48 to 3.68), and 12.82 (95% CI = 11.08 to 14.56), respectively. Summer was the only season exceeding the thermal comfort threshold.

### 3.2. The Oxidative Stress Analysis

**Figure 2:**
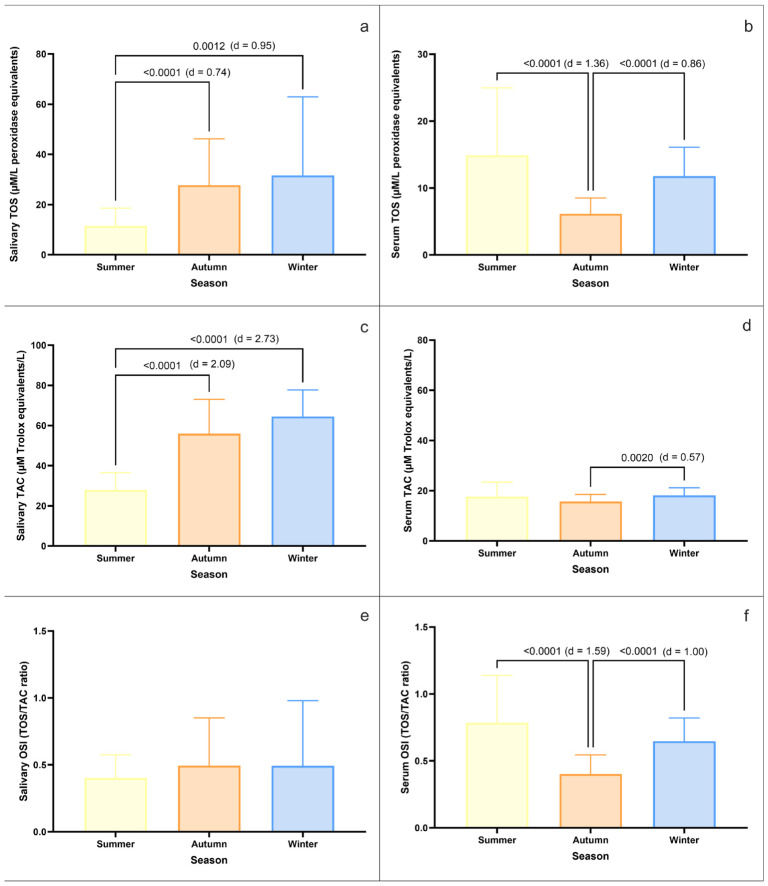
The concentrations of oxidative status biomarkers in the healthy dairy cows in summer, autumn and winter. Total oxidant status (TOS) in saliva (**a**) and serum (**b**), total antioxidant capacity (TAC) in saliva (**c**) and serum (**d**), and oxidative stress index (OSI) in saliva (**e**) and serum (**f**). The graphs show the means (boxes) and standard errors of the means (error bars). The horizontal brackets represent statistically significant differences between the groups; the level of significance is reported.

Assessing the results related to oxidative load in saliva, the lowest TOS concentration in the saliva samples was observed in summer, showing significant differences compared with autumn and winter, where higher oxidant values were detected (Figure 2a). The highest effect size was observed between summer and winter (d = 0.95, 95% CI = 0.49 to 1.42). In contrast, the highest TOS concentration was recorded in the serum samples in summer, followed by winter, with significantly lower values in autumn (Figure 2b). The comparison between autumn and summer showed a large effect size (d = 1.36, 95% CI = 0.88 to 1.84), whereas no significant differences were observed between summer and winter. In addition, winter showed significantly higher TOS concentrations than autumn (d = 0.86, 95% CI = 0.39 to 1.32).

Regarding antioxidant production, the salivary results showed a clear difference between summer, characterized by lower concentrations, and both winter and autumn, where TAC was higher (Figure 2c). Large effect sizes were observed for both comparisons between summer and autumn (d = 2.09, 95% CI = 1.56 to 2.61) and between summer and winter (d = 2.73, 95% CI = 2.16 to 3.30). In contrast, the serum samples showed significantly lower TAC concentrations in autumn compared with winter (Figure 2d; d = 0.57, 95% CI = 0.11 to 1.02), whereas no significant differences were observed between the other seasons.

The OSI ratio showed differences in the serum samples but not in saliva (Figure 2e,f). The summer OSI values in the serum samples differed from those in autumn, with an effect size of d = 1.59 (95% CI = 1.10 to 2.09). In addition, autumn differed significantly from winter (d = 1.00, 95% CI = 0.53 to 1.47) (Figure 2f). 

Descriptive statistics for all biomarkers are provided in Supplementary Table S1.

### 3.3. The Inflammatory Analysis

**Figure 3:**
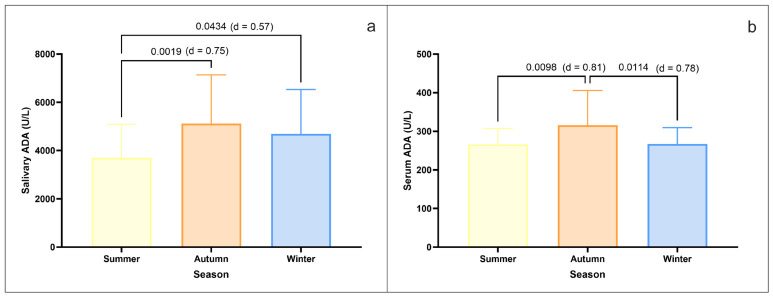
The measurement of adenosine deaminase (ADA) activity (U/L) in the saliva (**a**) and serum (**b**) samples in the healthy dairy cows in summer, autumn and winter. The graphs show the means (boxes) and standard errors of the means (error bars). The horizontal brackets represent statistically significant differences between the groups; the level of significance is reported.

In relation to ADA enzyme activity in the saliva samples, summer values were lower than the results from autumn and winter, with moderate effect sizes between summer and autumn (d = 0.75, 95% CI = 0.29 to 1.21) and between summer and winter (d = 0.57, 95% CI = 0.11 to 1.02) (Figure 3a). Summer demonstrated significant differences from the rest of the seasons. In parallel, ADA enzyme activity from the serum samples showed significant differences between summer and autumn, with higher concentrations observed in autumn and an effect size of 0.81 (CI = 0.35 to 1.27) (Figure 3b). Significant differences were also observed between autumn and winter (d = 0.78, 95% CI = 0.32 to 1.25), whereas no significant differences were detected between summer and winter.

The overall descriptive statistics of all the analyses performed in the saliva and serum samples in the different seasons are shown in Supplementary Table S1.

### 3.4. Productive and Reproductive Context

**Figure 4:**
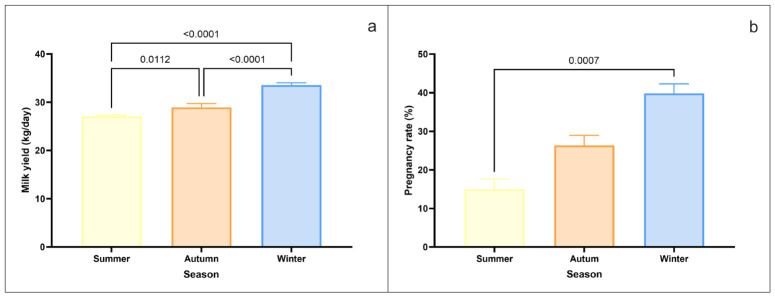
The milk yield values (**a**) and the pregnancy ratio (**b**) of the entire group of animals from the evaluated farm across summer, autumn and winter. The graphs show the means (boxes) and standard errors of the means (error bars). The horizontal brackets represent statistically significant differences between groups; the level of significance is reported.

Milk yield showed a 1.23-fold increase from the beginning of the study, summer, the season with the highest heat stress, to the end of the study, winter. Mean milk production values were 27.08 ± 0.13 kg/day in summer, 28.96 ± 0.36 kg/day in autumn and 33.55 ± 0.25 kg/day in winter. Significant differences in milk yield were observed among all the evaluated seasons (Figure 4a). 

The pregnancy rate in summer was 15.03%, whereas the values observed in autumn and winter were 26.35% and 39.83%, respectively, representing decreases of approximately 11 and 25 percentage points in summer compared with autumn and winter (Figure 4b). Statistical significance was only observed between summer and winter.

### 3.5. Correlation Between THI and Biomarkers in Saliva and Serum

When associations between THI and the studied parameters in saliva and serum were assessed (Table 1), statistically significant negative correlations were observed between THI and salivary TAC (strong; r = −0.72), TOS (moderate; r = −0.53), and ADA (weak; r = −0.23), whereas in serum, only TAC showed a weak negative association.

## 4. Discussion

It is known that heat stress has emerged as one of the most powerful challenges facing the dairy industry today [29], as milk yield decreased following exposure to heat stress [3].

The present study evaluated the effect of seasonal heat stress on oxidative and inflammatory status in healthy dairy cattle using serum and saliva biomarkers and shows that these responses are differentially reflected in both biological fluids. THI is used as an indicator of heat stress, and dairy cows are considered above the comfort threshold when THI exceeds 72 [30]. Other studies suggest that high-producing dairy cows may be negatively affected even at THI values lower than 72 [3,31]. We evaluated lactating cows with different mean THI values, specifically summer 79.30, autumn 58.74 and winter 53.49. Therefore, in our study, only individuals from summer were identified above this threshold, but in contrast, no animal was above the upper critical THI. When this limit is exceeded, the risk of death in dairy cows is maximum (THI > 87) [31]. Our summer THI values were similar to those reported in a study conducted in Serbia that evaluated the relationship between THI, milk production and feed intake in Holstein–Friesian cows across seasons [32]. However, the average daily THI that the researchers obtained in autumn was superior (66.36) and in winter was lower (42.34) than our values, showing the different external environmental conditions in both countries.

Milk yield is considered, next to dry matter intake, the performance parameter most affected by climatic variables [2]. We observed a decrease in milk yield in our evaluated animals in summer in comparison to the other two seasons of 1 and 1.2 kg, which is consistent with previous reports of heat-stress-related reductions in milk yield [33], whereas milk production progressively increased from autumn to winter. Moreover, a decrease in pregnancy rate between 11 and 25% in summer in comparison to the other two seasons studied was observed in our study and agrees with the longer calving-to-conception interval reported during summer in a subalpine region [34].

These productive and reproductive impairments are consistent with the presence of underlying systemic stress and homeostatic dysregulation induced by heat stress. Reduced performance, together with changes in immune and oxidative-related indicators, have been reported in heat-stressed dairy cows [4].

Seasonal thermal stress has also been associated with altered antioxidant status and impaired reproductive performance in dairy cows [34].

From a pathophysiological perspective, heat stress triggers physiological, endocrine and metabolic adjustments aimed at maintaining normothermia [5]. These systemic responses have been associated with changes in oxidative status, including alterations in oxidant production and antioxidant defences, which may contribute to oxidative stress when the imbalance is sustained [6]. Importantly, oxidative status and immune function are closely interconnected, and studies in dairy cows exposed to heat stress have described concurrent changes in oxidative biomarkers and inflammatory/immune-related indicators [4,6]. Therefore, integrating oxidant and antioxidant information through OSI provides a more informative interpretation of redox balance than considering either component alone [16] and offers an interpretative framework to contextualize the increased serum TOS and OSI observed during summer in the present study.

Regarding the analyses performed in the different body fluids, saliva was first evaluated to monitor the oxidative and inflammatory status of the cows across seasons, followed by serum analysis. Salivary TOS showed higher concentrations in the seasons with the lowest THI values, specifically autumn and winter. On the other hand, oxidant production was minor in summer, where THI was greater, and, therefore, a negative moderate association between both parameters was observed. These results of the higher oxidant production in the seasons with lower THI values coincided with those acquired in a previous seasonal study performed in serum [6]. In the cited study, higher lipid peroxidation was described at lower THI, whereas heat stress was associated with marked endocrine and immune alterations, indicating that oxidative responses may vary depending on environmental conditions and study design. Nevertheless, other studies report high plasma oxidant levels from cows subjected to heat stress for 50 days [4], which agree with our serum analysis. These discrepancies could be related to differences in experimental design among studies. However, the presence of high or low oxidant values alone is not a direct indicator of oxidative damage, which requires the consideration of antioxidant levels within an integrative index [16].

The values obtained for salivary TAC measurement showed a similar trend to the salivary TOS levels, evidencing a lower concentration in summer. This suggests higher antioxidant production in winter and autumn. Thus, increases in TOS were accompanied by rises in TAC, indicating a compensation mechanism to maintain oxidative balance, as observed in other studies [15]. This compensatory response is further reflected in the stable salivary OSI values across seasons. Although it has not been evidenced that there is oxidative stress in summer when observing salivary analytes, it may indicate that at the local level, the ability to balance the redox state is effective, and that shows valuable information. By contrast, the serum samples showed the highest oxidant levels in summer, while the TAC concentrations showed only minor seasonal variations, resulting in a high oxidative index in summer. Thus, unlike saliva, oxidative stress was evident in the serum samples under seasonal heat stress, with summer showing the highest oxidative imbalance, autumn the lowest, and winter intermediate values, as previously reported in summer compared to winter in the subalpine region [34]. Although the salivary results do not fully replicate the oxidative profile observed in the serum, previous studies in cattle reported that salivary oxidative parameters only partially reflect plasma oxidative status, possibly due to the local metabolism of the salivary glands [35]. The secretory activity of the salivary glands and the salivary composition in ruminants are influenced by multiple physiological factors, including rumination activity, resting period and chewing activity [17], as well as feeding behaviour and sampling-related physiological conditions [18], which could contribute to the different oxidative profiles observed between saliva and serum in the present study. In our study, we used the same protocol for saliva collection for all the animals and seasons to homogenize the possible influence on saliva composition due to these factors by sampling at the same time-range in the morning, at the specific moment just before feeding. However, other studies also report different oxidative responses in saliva and serum samples that should be further evaluated.

Heat stress has been reported to impair the immune function of dairy cows [36], including the activity of bovine lymphocytes in hot environments [37]. Consistent with these findings, we observed lower activity of the inflammatory biomarker, ADA, in summer in both saliva and serum. This is also in line with the low serum pro-inflammatory cytokine levels observed in cows subjected to heat stress [4] and compensatory high levels of anti-inflammatory cytokine [6]. The ADA levels observed in both body fluids are comparable to those reported in healthy cows in a previous article, in which higher ADA values were observed in diseased cows, with more intensity in animals with mastitis [13]. The increase in ADA levels has been highlighted next to the increase in the concentration of an acute phase protein, specifically haptoglobin, in a small pilot study about metritis in cows [20].

The productive and reproductive impairments observed during summer are consistent with the presence of systemic homeostatic challenges induced by heat stress. Oxidative stress and immune dysregulation have been proposed to contribute to the diversion of metabolic resources toward maintenance and cellular protection, thereby reducing the energy available for milk synthesis and reproductive processes [2,4,34]. In addition, inflammatory and oxidative mediators can impair ovarian function and the uterine environment, contributing to reduced fertility under hot conditions [29]. Although causal relationships cannot be established in the present observational design, the parallel occurrence of increased serum OSI, reduced ADA activity, and lower milk yield and pregnancy rates during summer is consistent with a biologically plausible link between systemic redox–immune imbalance and impaired performance.

From a clinical and herd-management perspective, the present findings suggest that monitoring serum oxidative and immune-related biomarkers may contribute to the assessment of heat-stress-associated homeostatic disturbances in dairy cows. In particular, increases in serum OSI together with reductions in ADA activity could help identify animals experiencing systemic physiological strain during hot periods. However, these biomarkers should not be considered standalone diagnostic tools, and their interpretation requires integration with environmental indices such as THI, productive parameters, and clinical evaluation. Further longitudinal and multi-farm studies are required before recommending their routine use for decision-making under field conditions.

Several limitations of the present study should be acknowledged. First, the observational and seasonal design precludes causal inference between heat stress and the redox–immune alterations observed. Second, the animals were sampled from a single farm, which may limit the generalizability of the findings to other production systems or climatic regions. In addition, the cows were not stratified according to stage of lactation or days in milk, factors that may modulate oxidative and immune responses to thermal stress. Finally, the lack of circulating cytokine measurements restricts a more detailed characterization of immune pathways involved in the observed responses. Future multi-farm longitudinal studies incorporating broader immunological and metabolic profiling are warranted to validate and extend these findings.

Together, these findings suggest that seasonal heat stress is associated with a shift toward reduced immune activation rather than activation in healthy dairy cows, which may contribute to their increased susceptibility to disease under hot conditions.

Following the evaluation of the different analyses performed in both biological fluids, we conclude that the two biological fluids exhibited different response profiles. Therefore, serum analysis appears to be the most appropriate tool for assessing heat-stress-related health and welfare alterations in dairy cows, based on oxidative status and ADA measurements, under the conditions and biomarkers evaluated in the present study. Nevertheless, no studies have yet compared the time course of the oxidative stress in serum and saliva samples, which should be investigated in further studies to evaluate the relationship between salivary oxidative stress biomarkers and heat stress.

It is crucial to consider that strategies aimed at increasing milk yield may make dairy cows more sensitive to heat stress, unless tolerance is achieved through genetic selection [3]. Currently, to the best of our knowledge, there is no description of morbidity and mortality pathogenesis associated with heat stress in dairy cows [38]. However, a relationship has been observed between seasonal effects and animal mortality, with a greater death rate in summer, i.e., when THI is the highest (THI > 87) [31]. Thus, an easy detection of the homeostasis dysregulations related to heat stress would be of great value, and in this challenge, measuring the oxidative stress and immune impairment biomarkers, as reported in the present study, could provide an effective approach.

## 5. Conclusions

This study concludes that seasonal heat stress (THI > 79) triggers a state of systemic redox imbalance and immune suppression in dairy cattle. Serum analysis, specifically through the OSI and ADA activity, proved to be a more reliable diagnostic tool than saliva for detecting these homeostatic disturbances. Although salivary biomarkers exhibit seasonal sensitivity, they do not fully mirror the systemic oxidative response observed in serum, likely due to local glandular metabolism. Consequently, serum appears to be the most appropriate biological fluid for assessing the impact of heat stress under the conditions evaluated in the present study. These results underscore the importance of monitoring multi-marker profiles to mitigate the reproductive and productive losses associated with thermal stress in Mediterranean dairy systems.

## Figures and Tables

**Table 1:** Correlation analysis between THI and TOS, TAC, OSI and ADA in saliva and serum samples.

Parameters	r	95% Confidence Interval	*p* Value
**Saliva**			
TOS	−0.53	−0.66 to −0.38	<0.0001
TAC	−0.72	−0.80 to −0.61	<0.0001
OSI	−0.06	−0.25 to 0.12	0.4963
ADA	−0.23	−0.40 to −0.048	0.0140
**Serum**			
TOS	−0.07	−0.26 to 0.128	0.4582
TAC	−0.23	−0.40 to −0.04	0.0142
OSI	0.05	−0.14 to 0.23	0.5959
ADA	0.02	−0.17 to 0.21	0.8073

TOS, total oxidant status; TAC, total antioxidant capacity; OSI, oxidative stress index; ADA, adenosine deaminase; THI, temperature–humidity index.; r, Spearman coefficients of correlations.

## Data Availability

Dataset available on request from the authors.

## Supplementary Materials

The following supporting information can be downloaded at https://www.mdpi.com/article/10.3390/ani16121758/s1, Supplementary Table S1. Descriptive statistics of TOS, TAC, OSI and ADA in saliva and serum samples obtained from dairy cows in summer (n = 38), autumn (n = 38) and winter (n = 38).

## Abbreviations

The following abbreviations are used in this manuscript:

THI	Temperature–humidity index
OSI	Oxidative stress index
TAC	Total antioxidant capacity
TOS	Total oxidant status
ADA	Adenosine deaminase
T	Temperature ambient
RH	Relative humidity
FRAP	Ferric reducing ability of plasma
