# Supplementary material for: Systemic Oxidative and Inflammatory Responses to Seasonal Heat Stress in Dairy Cattle: Comparison of Serum and Saliva Biomarkers
Source: Animals (Basel). 2026 Jun 6;16(12):1758. doi: 10.3390/ani16121758 (PMC13295259; doi:10.3390/ani16121758)
Supplement: Supplementary file 1 [file animals-16-01758-s001.zip › animals-4329128-supplementary.pdf]

Supplementary Table S1. Descriptive statistics of TOS, TAC, OSI and ADA in saliva and serum samples obtained from dairy cows in summer (n = 38), autumn (n = 38) and winter (n = 38).

	Biological fluid	
	Saliva	Serum
**Parameters**[1)]	Mean (SEM)	Mean (SEM)
**TOS**		
Summer	11.5 (± 1.151)	14.87 (± 1.640)
Autumn	27.74 (± 3.002)	6.151 (± 0.383)
Winter	31.6 (± 5.080)	11.78 (± 0.701)
**TAC**		
Summer	27.90 (± 1.409)	17.62 (± 0.942)
Autumn	55.96 (± 2.777)	15.66 (± 0.459)
Winter	64.74 (± 2.154)	18.07 (± 0.501)
**OSI**		
Summer	0.402 (± 0.028)	0.7845 (± 0.057)
Autumn	0.4940 (± 0.0578)	0.4015 (± 0.023)
Winter	0.4922 (± 0.079)	0.6462 (± 0.028)
**ADA**		
Summer	3702 (± 225)	266.7 (± 6.537)
Autumn	5122 (± 326.7)	316 (± 14.58)
Winter	4689 (± 298.6)	267.2 (± 6.896)

SEM, standard error of the means. TOS, total oxidant status. TAC, total antioxidant capacity. OSI, oxidative stress index. ADA, adenosine deaminase

[1)]TOS (μM/L peroxidase equivalents); TAC (μM/L Trolox equivalents); OSI (TOS/TAC ratio); ADA (U/L)
